# Spatial omics strategies for investigating human carotid atherosclerotic disease

**DOI:** 10.1002/ctm2.70277

**Published:** 2025-03-28

**Authors:** Jiaxin Wan, Zhi Sun, Xueqiong Feng, Peipei Zhou, Mateus T. N. Macho, Zhouyang Jiao, Hui Cao, Chuang Zhang, Rijin Lin, Xiaowen Zhang, Mengyan Fan, Nan Zhang, Jiamei Zhang, Huixiang Liu, Jing Li, Sheng Guan

**Affiliations:** ^1^ Department of Neurointervention the First Affiliated Hospital of Zhengzhou, University Henan Provincial Neurointerventional Engineering Research Center Zhengzhou Henan China; ^2^ Department of Pharmacy the First Affiliated Hospital of Zhengzhou University, Zhengzhou, China. Henan Engineering Research Center of Clinical Mass Spectrometry for Precision Medicine Zhengzhou Henan Province China; ^3^ Department of Cardiovascular Surgery The First Affiliated Hospital of Zhengzhou University Zhengzhou China; ^4^ Department of Endovascular Surgery The First Affiliated Hospital of Zhengzhou University Zhengzhou China; ^5^ Department of Emergency Medicine The First Affiliated Hospital of Zhengzhou, University Zhengzhou China

**Keywords:** atherosclerosis, biomarker, spatial distribution, spatial omics, vascular bed

## Abstract

Atherosclerosis is a chronic inflammatory condition of the arteries, marked by the development of plaques within the arterial intima. The rupture of unstable plaques can lead to thrombosis, downstream vessel occlusion and serious clinical events. The composition of atherosclerotic plaques is complex and highly heterogeneous, posing challenges for their study. The current pathology and histological subtype classification of plaques may fail to fully encompass the microscopic molecular components in the tissue, the disease progress in various stages of atherosclerosis and the potential mechanism of plaque rupture. However, spatial mapping of the heterogeneity in plaque tissue components can enhance our understanding of these lesions. Despite the considerable progress made by traditional omics in the field of disease research, and its status as an indispensable technology, there remain inherent limitations in the investigation of minute molecular. In recent years, spatial omics techniques have advanced significantly, enabling the visualisation and analysis of specific components within plaques that may serve as causal targets associated with disease progression. The effective application of spatial omics in both research and clinical settings represents a promising area for further exploration. This review focuses on the recent advancements and findings related to spatial omics in the study of extracranial carotid atherosclerotic cerebrovascular disease. Spatial omics analysis of atherosclerotic plaques can facilitate the detection of biomarkers with diagnostic significance or potential relevance to disease, offering new methods and insights into the diagnosis of atherosclerosis and its complications.

## Graphical abstract

Spatial omics, by facilitating visualised precision medicine and accomplishing the spatial localisation of microscopic substances, is poised to emerge as a crucial tool in future research areas, including the exploration of molecular pathways, the implementation of precision targeted therapies and the refinement of disease diagnostic techniques.

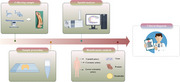

## 1 INTRODUCTION

Worldwide, stroke continues to be the second most frequent cause of mortality and the third most common cause of disability.^1^ All population groups are at risk for stroke. The disease should no longer be limited to the elderly, as one‐third of those affected by stroke are aged under 65 years. Moreover, extracranial carotid artery disease accounts for approximately 15% of all cases of cerebral ischaemia.^2^ There are over 50 million survivors of stroke and transient ischaemic attack; however, more than 20% of these individuals are likely to experience a recurrent stroke within the next 5 years, significantly increasing their risk of disability.^3^ Approximately 80% of strokes are ischaemic.^4^ About 20% of these strokes are caused by atherosclerosis of the large arteries.^5^

Atheromatous plaque can form anywhere in the carotid arteries, but it most commonly occurs in the internal carotid arteries.^6^ Strokes of this type are known as atherosclerotic strokes and have a higher recurrence rate than other types.^7^ Atherosclerosis is a chronic inflammatory disease that occurs in the walls of the arteries^8^ and is closely related to lipids. As the lesion progresses, it can lead to thickening and hardening of the arteries, decreasing the flow of blood through the lumen, and rupture or erosion of the blood vessels in the area of the lesion can occur.^9^ However, when a plaque suddenly ruptures, it can create a thrombus within the vessel that travels with the blood flow, resulting in occlusion of the vessel at a particular site. Rupture of atherosclerotic plaques formed at the carotid arteries creates characteristic cerebrovascular disease that can promote ischaemic events in the brain. The aetiology of carotid atherosclerosis is still unclear, its pathogenesis is complex and the disease might be related to genetic components, chromosomal anomalies, gene fusions and further aspects.^10^ Therefore, it is important to study in depth the specific mechanisms of carotid atherosclerosis and the structure of its components in the context of atherosclerotic stroke disease. Furthermore, the disease involves multiple vascular beds, and plaques formed at different sites exhibit disparate clinical manifestations and pathologic features.^11^ Thus, accurately determining the precise location of these differences remains a scientific challenge.

Despite the fact that the traditional approach can detect a large number of microscopic molecules, its limitation is that it does not allow a clear perception of the specific localisation of the detected substances. We need methods to objectively understand the spatial molecular changes in these pathologic changes over a period of time or at a moment in time. The increasing maturity of spatial omics technology can well make up for the shortcomings of traditional methods. The heterogeneity of plaque components can be reflected in different pathological periods, and the complex co‐interaction of various components at the molecular level as well as their mutual regulation can be detected by spatial technology. This promising tool opens up new perspectives for microscopic molecular studies.

This review summarises the advances in spatial omics in the study of carotid atherosclerosis disease (Table 1) and the emerging applications of the study tools (Table 2), as well as the advantages of spatial omics for studying this disease. Finally, potential directions for future atherosclerosis research efforts will be discussed.

**TABLE 1:** Current progress in carotid atherosclerosis spatial omics research.

Spatial omics	Technology	Sample	Evidence	Years	References
Spatial transcriptomics	10x Genomics‐Visium	Human	The cloning region of AS: ACTA2↑, CD74↓, CTSB↓, HLA‐DPA1↓, SERF‐2↓.	2023	^71^
	10x Genomics‐Visium	Human	Localised the distribution of mRNA levels of MMP9, IGKC and PLN.	2023	^73^
	ISH	Human, mouse	SMC specific expression of Itih4 in lesions.	2024	^74^
Spatial proteomics	CODEX	Human	Bilirubin spatially correlates with the cytoplasmic content of inflammatory macrophages carrying CD74, HLA‐DR, CD14 and CD163 markers.	2023	^84^
Spatial metabolomics	DESI‐MSI	Human	SM, Cer, PC and LysoPC may be novel potential targets.	2023	^93^
	DESI‐MSI	Human	Reveals several molecular species co‐localised with plaque‐associated disease processes, including SM, Cer, FFA, phospholipids, triacylglycerols and phosphatidylinositol.	2023	^94^
	DESI‐MSI	Human	The presence of sodium and chloride adducts was identified in diacylglycerophosphocholines (GPChos), SMs and hydrolysed GPChos.	2009	^65^
	MALDI‐MSI	Human, mouse	LPC and LPA were significantly elevated in advanced plaque.	2024	^96^
	MALDI‐MSI	Human	Regions of macrophages in symptomatic lesions are enriched in sphingolipids and endothelial vascular smooth muscle cells are enriched in cholesterol and cholesteryl esters.	2021	^58^
	MALDI‐MSI	Human	Sphingolipids and oxidised cholesterol esters correlate with regions of necrotic endosomes. Correlation of diacylglycerol and triacylglycerol with regions containing the coagulation protein fibronectin.	2021	^92^

**TABLE 2:** Tools available for the study of spatial omics.

Spatial omics	Main technologies	Resolution	Study Type	Reference
Spatial transcriptomics	**Sequencing‐Based Technologies**:			
	10x Genomics‐Visium	∼55µm/spot	RNA	^21^
	Slide‐seq	∼10µm/spot	RNA/DNA	^22^
	DBiT‐seq	∼10µm/pixel	RNA/pro	^23^
	Light‐seq	Close to single cell	RNA	^25^
	Stereo‐seq	Close to single cell	RNA	^24^
	**Imaging‐Based Approaches**:			
	HybISS	x	RNA	^27^
	FISSEQ	x	RNA	^28^
	seqFISH	x	RNA/pro	^30^
	seqFISH+	x	RNA/pro	^31^
	merFISH	x	RNA/pro	^32^
Spatial proteomics	NanoSIM	∼40nm	lipid/metabolite/pro	^44^
	MALDI‐MSI	10∼100µm/pixel	lipid/metabolite/peptides/pro	^45^, ^46^
	4i	165 x 165nm/pixel	pro	^36^
	IBEX	x	pro	^37^
	CODEX	x	pro	^39^
	Immuno‐SABER	x	pro	^41^
Spatial metabolomic	SIMS‐MSI	∼1µm	lipid/small molecules	^53^
	MALDI‐MSI	10∼100µm/pixel	lipid/metabolite/peptides/pro	^63^
	DESI‐MSI	∼200µm	lipids/metabolite/small peptides	^68^

## 2 PROGRESSING SPATIAL OMICS

In recent decades, omics has emerged as a crucial concept across various research domains, significantly influencing the field of atherosclerosis and marking a pivotal shift in our understanding of this condition. Traditional omics approaches – such as genomics, transcriptomics, proteomics, metabolomics and lipidomics – provide an extensive depiction of the molecular alterations within atherosclerotic plaques.^12^ Among these, spatial omics is increasingly recognised as a new frontier in life sciences,^13^ integrating the physical structure of tissues with their molecular features and promising to transform many biological aspects while revolutionising conventional perspectives on disease.

Spatial omics enhances traditional omics by adding a spatial dimension, enabling more efficient observation and selection of specific regions for study and analysis. These approaches are characterised by high spatial resolution, extensive coverage, adaptability in size, high throughput and multiplexing capabilities.^14^

Despite these advances, existing techniques for detecting atherosclerotic plaques have notable limitations. Therefore, there is a pressing need for new methods and concepts to uncover novel mechanisms and therapeutic targets, as well as to identify biomarkers that can more effectively assess risk. This is essential for improving medical decision‐making in cases of atherosclerotic stroke. Spatial omics presents a promising solution by addressing these research gaps.

**FIGURE 1:**
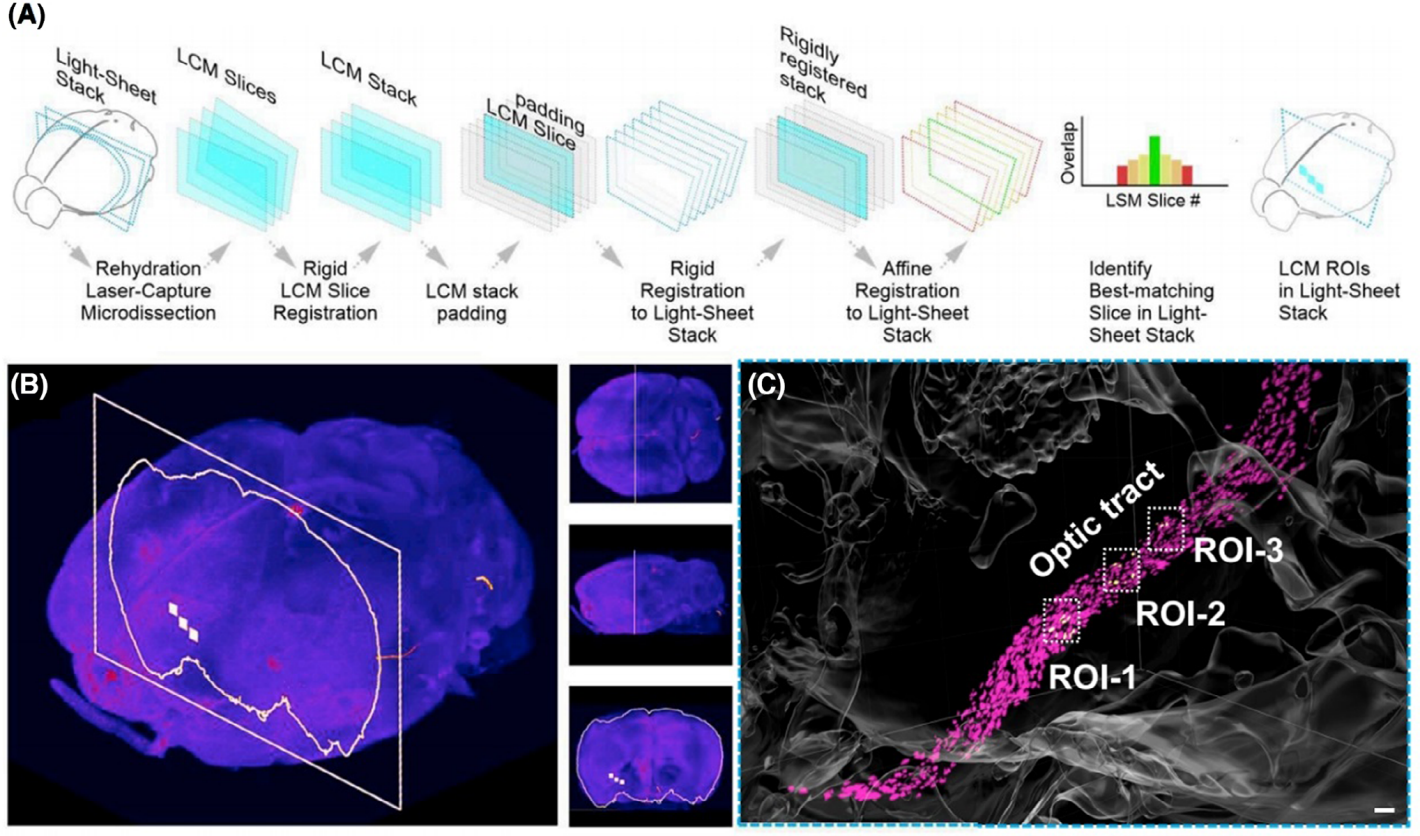
Proteomes of micro‐dissected tissues imaged in 3D. Using a mild traumatic brain injury (mTBI) mouse model to identify and analyse the proteome of brain regions containing discrete local inflammation.^16^ (A) Automatically register 2D laser capture microdissection images back to the 3D light sheet imaging stack. (B) LCM slice location within the stack, the ROI is then projected onto the 3D light sheet imaging data. (C) 3D reconstruction of the digitally stitched images revealed a significant increase in the number of activated microglia (magenta) along the region of the optic beam.

As spatial omics technology continues to be applied across various fields, it is poised to play a critical role in reconstructing spatial layouts in future research. Currently, spatial omics functions as an approximation of 3D profiling, primarily because most experiments are conducted on thin tissue sections. While these sections can be obtained at the appropriate thickness, analyses are typically limited to their surfaces, resulting in a 2D perspective that does not fully capture the complexities of 3D space. Looking ahead, advancements in spatial omics technologies and the introduction of innovative research processes will enhance research precision, paving the way for the development of diverse 3D analysis methods and techniques (Figure 1).^15^, ^16^, ^17^, ^18^

## 3 SPATIAL OMICS RESEARCH STRATEGIES FOR CAROTID ATHEROSCLEROSIS

### 3.1 Main techniques of spatial transcriptomics

Spatial transcriptomics (ST) technologies can be broadly categorised into sequencing‐based and imaging‐based. Notably, different spatial transcriptome approaches focus on different features in terms of genes captured, resolution and overall transcriptome assessment, and spatial omics methods and technologies are upgraded continuously and implemented to meet the needs in terms of gene or protein detection, sensitivity, resolution, ease of manipulation and region size. By means of a systematic approach to measuring gene expression levels across the tissue space, we were able to elucidate the underlying interactions and phenotypes within tissues.

#### 3.1.1 Sequencing‐based technologies

Sequencing and next‐generation sequencing (NGS) methods are effective research tools that can detect RNA transcription products to reveal biological mechanisms and genetic differences.

Sequencing‐based approaches depend on whole transcriptomes based on spatially barcoded DNA, using barcoded DNA arrays to capture polyadenylated RNA transcripts from tissues, which can then be subjected to NGS. The NGS technology is a high‐throughput sequencing technology capable of rapidly and accurately determining a large number of DNA or RNA sequences in a brief span of time,^19^ and our spatial omics using the NGS method allows genome‐wide and transcriptome‐wide capture to provide high‐throughput gene expression data. Spatially resolved transcriptome analysis is achieved by capturing mRNA in tissue sections using a barcoded solid matrix, enabling unbiased whole transcriptome analysis using poly(dT) oligonucleotides to capture target sequences on spatial barcode arrays with poly(A) tails and correlating transcripts with their spatial locations.^20^

**FIGURE 2:**
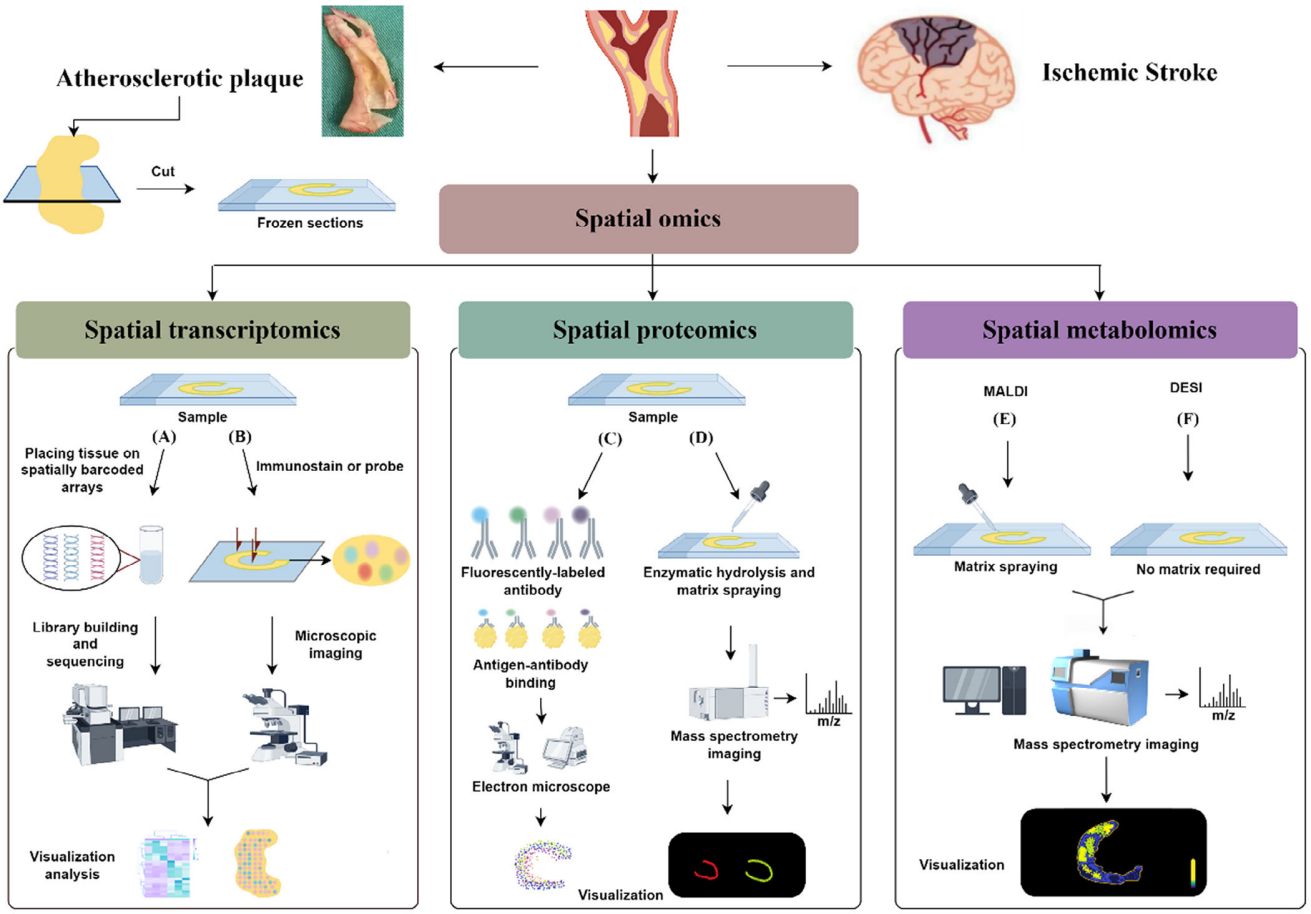
Application of spatial omics in atherosclerosis. (By Figdraw.) Plaque samples stripped from the intima of vessels were processed for frozen sections. (A) Placing tissue on spatially barcoded arrays and then captured for sequencing of adenylated RNA, which was mapped back to its original locus by reverse transcription to demonstrate its enrichment in specific regions. (B) Visualisation and analysis by microimaging after in situ hybridisation of samples using immunostain or probe technology. (C) Use fluorescently labelled antibodies to label target proteins and take advantage of the properties of fluorescence to visualise the spatial distribution of proteins through fluorescence microscopy. (D) Samples were enzymatically digested and matrix sprayed, and sent to the MALDI device for detection, obtaining mass spectra and corresponding in situ distributions. The difference between MALDI and DESI is that MALDI (E) requires matrix spraying, whereas DESI (F) does not, and the spatial distribution of the substance is obtained by processing the equipment.

10x Genomics recently launched a new technology called Visium, which enables large‐scale tissue detection without pre‐selecting the region of interest (ROI). It analyses the entire transcriptome from tissue sections by capturing polyadenylated RNA on spatially barcoded microarray slides (Figure 2A). At this stage, Visium has a higher resolution and sensitivity(capture >10^4^ transcripts per spot).^21^ The method is suitable for experiments that require sectioning.

In order to obtain complete spatial information and morphology (especially in tissues), current ST approaches have used various DNA barcoded beads to obtain valid information. Slide‐Seq is an additional NGS‐based technique; under the same area of the region, this method has higher resolution and sensitivity compared with Visium.^22^ The DBiT‐Seq (Deterministic Barcoding in Tissue for spatial omics sequencing) method, which uses microfluidics to apply polyT barcodes to tissue sections, enables spatial omics sequencing at 10 µm pixel size.^23^ Stereo‐seq (spatio‐temporal enhanced resolution omics sequencing) is a novel approach using arrays of randomly barcoded DNA nanoballs for nanoscale resolution. It can expand the scope of detection, and applications are already underway.^24^

Light‐Seq, another NGS‐based spatial genomics technology, is a method for multiple spatial indexing of intact biological samples in fixed cells and tissues using light‐guided DNA barcodes. This light‐guided DNA barcoding allows for in situ selection of multiple cell populations within intact and fixed tissue samples, without the need for cell dissociation and is suitable for use in whole transcriptome sequencing on the basis of positional, morphological and protein staining.^25^

Consequently, the spatial technology of sequencing enables the measurement of gene expression, the detection of mutations and variants, an in‐depth analysis of spatial differences at the genetic level and the implementation of individual treatments for diseases.

#### 3.1.2 Imaging‐based approaches

The research approach to ST imaging has been reported to be based on two aspects: in situ sequencing (ISS)‐based methods and in situ hybridisation (ISH)‐based methods.^26^

Imaging‐based techniques employ the iterative hybridisation of fluorescently tagged probes combined with sequence imaging to attain single‐molecule resolution of in situ gene profiles.^20^ Both ISH and ISS are commonly used research tools with high spatial resolution and the capability to detect mRNAs at the subcellular level (Figure 2B).

The ISS‐based method directly reads the sequence of transcripts in tissues. the development of HybISS has improved detection efficiency, resulting in increased read lengths, improved throughput and the ability to use cellular barcodes.^27^ For non‐targeted analysis of ISS, FISSEQ can be used,^28^ and the current approach of combining FISSEQ and amplification microscopy can effectively avoid the problems of optical crowding and reduced sensitivity that can result from the non‐targeting method of amplification.^29^

The ISH‐based methods are performed using ISH techniques in which target sequences are detected by hybridising to complementary fluorescent probes. Currently, commonly used ISH methods include seqFISH,^30^ seqFISH+^31^ and merFISH.^32^ All of these methods perform multiple rounds of hybridisation and imaging, expanding the original single‐molecule fluorescence ISH. The merFISH performs successive rounds of hybridisation and imaging by combining fluorescence ISH with combinatorial labelling techniques. Sequential fluorescence ISH (seqFISH and seqFISH+) is a method to increase the number of genes detected at subcellular resolution through the combination of colours to pseudocolours. This method is commonly used to study intracellular organisation and is similar to merFISH, allowing the detection of a greater number of distinguishable transcripts.^33^

ST techniques offer an accurate representation of spatial composition and have been employed to generate maps of pathological areas of tissue. The ST‐based approach enables the precise location of the transcriptional profile across the aim region, thereby facilitating the identification of the transcriptomic signature of the lesion area.

### 3.2 Main techniques of spatial proteomics

Ongoing technological innovations and advances have made spatial proteomics techniques more flexible and efficient, which offers broader comprehensive information to study the spatial arrangement and protein–protein interactions within tissues and cells. Immunohistochemistry (IHC), immunofluorescence (IF) and mass spectrometry (MS) are the primary techniques in proteomics for analysing protein distributions.^34^

#### 3.2.1 Fluorescent antibody‐based approaches

The origins of imaging in spatial proteomics can be traced back to the visualisation of individual proteins through IF analysis. IF is a technique that utilises fluorescently labelled antibodies to visualise the spatial distribution of target proteins through fluorescence microscopy. It is a highly sensitive method with the ability to perform multicolour staining, making it a significant approach for studying protein subcellular localisation and interactions (Figure 2C).^35^ However, when using IF technology for intracellular staining, the fixation and permeabilisation processes may generate artifacts that impact cell morphology and protein localisation. This is a critical concern during experimental procedures.^34^ Another method, IHC is a rapid and straightforward approach that allows for the investigation of protein expression at the single‐cell level within intact tissue environments. It uses specific antibodies to bind to target proteins in tissues and visualise the location and expression levels of the target proteins through staining reactions. Nonetheless, in order to ensure the veracity of the IHC results, it is necessary to implement rigorous procedures and appropriate antibody validation strategies.^13^

Furthermore, the advancement of fluorescence‐based technologies, such as iterative indirect IF imaging (4i) and iterative bleaching extends multiplexity (IBEX), as well as co‐detection by indexing (CODEX) and DNA oligo‐labelling in immunostaining with signal amplification by exchange reaction (Immuno‐SABER), a variety of technologies with different types of protocols help researchers to localise the spatial distribution of proteins (Table 2). The 4i technique enables the visualisation of numerous proteins in thin tissue sections at high resolution through multiplexed indirect IF protocols, a feat that conventional techniques often struggle to achieve. However, the entire 4i technology process may necessitate protracted processing times, during which intricate operations and specialised equipment are requisite.^36^ IBEX is an open‐source approach that combines immunolabelling and chemical bleaching of fluorophores. This imaging technique can be performed in short cycles at a relatively low cost and offers a broad range of compatible antibody protocols. Furthermore, it has been demonstrated to enable the expeditious acquisition of complex imaging datasets from a diverse range of biological tissues.^37^, ^38^ CODEX spatial proteomics platform based on unique protein fluorescent labelling can well resolve the complex cellular composition and spatial distribution of proteins within the tissue microenvironment, and the core of CODEX's technology is the specific DNA Barcode labelled on each antibody. The fluorescent moiety binds specifically to the antibody that has been labelled with the corresponding Barcode. When the antibodies are fully conjugated to their respective antigenic targets, specific protein targets can be identified through the combination of the Barcode and the fluorescent moiety on the antibody for rapid and high‐throughput imaging analysis.^39^, ^40^ The Immuno‐SABER technique is a DNA‐based signal amplification method that amplifies fluorescent signals by using DNA‐barcoded antibodies and cyclic hybridisation of fluorescently labelled imaging strands, enabling highly multiplexed imaging of biological tissues. Nevertheless, the technique requires multiple cycles of hybridisation of fluorescent imaging strands with DNA‐barcoded labelled antibodies, which may increase the complexity and cost of the experiment.^41^ However, this repetitive cycle is eliminated in the SABER‐IMC method, which in turn improves the efficiency of the experiment.^42^

#### 3.2.2 MS‐based approaches

Although antigen–antibody binding is remarkably useful in various applications, it falls short in exploring cellular heterogeneity and disease mechanisms when compared with certain alternative techniques. MS‐based spatial proteomics technology can make up for the shortcoming of targeted detection of the spatial distribution of target proteins of interest and validation and is suitable for large‐scale protein detection and analysis. As an alternative to antibody‐based methods, the combination of MS and tissue sectioning allows for in situ quantitative analysis and imaging of proteins in tissues. The rapid acquisition of spatial localisation of proteins from tissue samples is enabled by mass spectrometry imaging (MSI), which integrates the MS technique with imaging methodologies. This integration of MSI facilitates a significant enhancement in the speed of analysis when compared with conventional techniques. The identification of proteins relies on the sequence of their unique peptides. The workflow encompasses protein extraction from tissue samples, enzymatic digestion of proteins using trypsin and subsequent separation of the resulting peptides via MS, the MS signal is then subjected to analysis by a spectrometer and dedicated image analysis software.^43^

NanoSIMS is an extension of secondary ion mass spectrometry (SIMS) that further improves resolution, allowing visualisation of subcellular structures; however, it is restricted to the analysis of microscopic molecules.^44^ Within spatial proteomics research, matrix‐assisted laser desorption ionisation (MALDI) serves as a useful analytical method (Figure 2D).^45^, ^46^ With the goal of characterising as many small molecules as possible, MALDI is a promising technique for spatial omics analysis. Recently, Claes et al.^47^ introduced a new technology that takes advantage of MALDI‐MSI and IHC to achieve highly multiplexed and targeted imaging of biomolecules within tissues. This can compensate for the shortcomings of protein detection in non‐targeted methods, thereby enabling a more comprehensive identification of proteins.^47^

### 3.3 Main techniques of spatial metabolomics

Metabolomics research plays a key role in identifying new therapeutic targets for diseases and discovering biomarkers. The combination of metabolomics and spatial omics has enabled scientists to gain insights into how the molecular distribution of metabolites can significantly impact the biology of diseases, addressing the challenge of poorly localised metabolites in tissues. Spatial metabolomics is concerned with detecting and interpreting metabolites, lipids, drugs and other small molecules in cell, tissue, organ and organism environments.^48^ With the development of MSI technology, which can target lesions more precisely for future studies, the rise of this technology has also successfully contributed to a new trend in the field of atherosclerosis research. Alterations in the spatial distribution of small molecules are directly related to the anatomical distribution of biological information, and MS provides an important way to characterise the abundance of molecules in tissues.

MSI is a molecular imaging method based on MS analysis technology. Following the requisite steps of preparation, after ionisation, the sample molecules are introduced into the mass spectrometer, and then they are separated according to their mass‐to‐charge ratio (*m*/*z*) values through the influence of an electric or magnetic field. The mass spectrometer is capable of determining the mass‐to‐charge ratio of the ions by measuring the time of flight, orbital radius and other parameters, thus obtaining the mass spectral information of the different molecules in the sample.^49^ The mass spectrometer, under the control of the software, scans the sample in accordance with the predefined parameters and records mass spectral data at each scanning point. These data comprise ion intensities at differing mass‐to‐charge ratios, which reflect the molecular composition and abundance of the sample at that particular point. Subsequent to conversion of the MS data into pixel points on the basis of the set *m*/*z* range and abundance parameters, the image is reconstructed and analysed.^50^, ^51^, ^52^ This leads to formation of a MSI map reflecting the spatial distribution of the molecules in the sample.

The use of MALDI and SIMS in spatial analysis has proven to be a highly efficacious approach.^53^ However, it should be noted that the prerequisites for these experiments are relatively stringent, necessitating desorption and ionisation in a vacuum environment, in addition to meticulous sample preparation, a process that often demands a considerable investment of time. In contrast, recent advancements in the field, namely desorption electrospray ionisation (DESI) has facilitated the development of high‐throughput MS methods. A notable advantage of DESI is that it improve the need for complex sample pre‐treatment, thus enabling more rapid analysis of samples under atmospheric conditions.^54^

#### 3.3.1 Secondary ion mass spectroscopy mass spectrometry imaging

Time‐of‐flight secondary ion mass spectrometry (TOF‐SIMS) is a broadly used method to detect, identify and localise targets on tissue sections with spatial resolution down to <1 mm. Compared with DESI and MALDI, high spatial resolution is an outstanding feature of SIMS for imaging tissue samples.^55^ In fact, the SIMS experiment can be either static or dynamic, with static mode being used almost analyses lipids exclusively.^56^ Simultaneous ion beam exposure of 1% of surface molecules was observed under static SIMS conditions. This minimises the number of repeated analyses of the same region, with the advantage that each new region is not damaged by previous beam collisions. By doing so, molecular ions will be more likely to be detected. However, even under these conditions, molecular fragmentation is often caused by high energy resolution. This means that intact components are poorly observed with atomic ion sources. For profiling experiments, it is more appropriate to use the softer MALDI and DESI techniques. In spite of this, SIMS is a uniquely powerful molecular analysis method, allowing it to provide spatially resolved molecular information at submicron resolution (in contrast with laser‐based methods that have typically 25–200 µm resolutions, like spectroscopy).^53^

Moreover, TOF‐SIMS analysis can also be used to analyse larger regions, but tiling artifacts are a common challenge after imaging, and the effectiveness of artifact removal methods depends largely on the details of the data set being analysed, so how to better remove data bias caused by artifacts and improve the utility of SIMS when analysing larger regional samples using TOF‐SIMS is also a future research trend.^57^

#### 3.3.2 Matrix‐assisted laser desorption ionisation mass spectrometry imaging

MALDI, the most frequently used technique for the direct detection of lipids from surfaces, is equally useful for the analysis of peptides, proteins and glycans and requires the use of different MALDI matrices and tissue preparation procedures in experiments as appropriate.^58^ However, careful matching of the matrix (particularly chemically relevant solvents) to the analyte of interest is critical (Figure 2E).^59^ The ability of MALDI‐MSI to identify and visualise hundreds of components directly from tissues in a label‐free and untargeted manner, while documenting the distribution of hundreds to thousands of molecules, has led to an increasing number of applications in atherosclerosis research.^60^ MSI begins by acquiring a spatially resolved MS‐based image and then computes an image representing the distribution of the analyte based on the intensity of the mass spectral peaks, allowing the biochemistry of the target to be studied directly through tissue analysis.^61^ MALDI‐MSI has been applied to a diverse array of tissues in a multitude of biological settings successfully. For example, to analyse the altered lipid content and spatial distribution that exists in diseased tissues. The ability to acquire spectra conveniently in a brief span of time is also a key advantage of MALDI‐ MSI. Basically, many classes of lipids can be the subject of MALDI‐MS analysis and investigation.^59^

**FIGURE 3:**
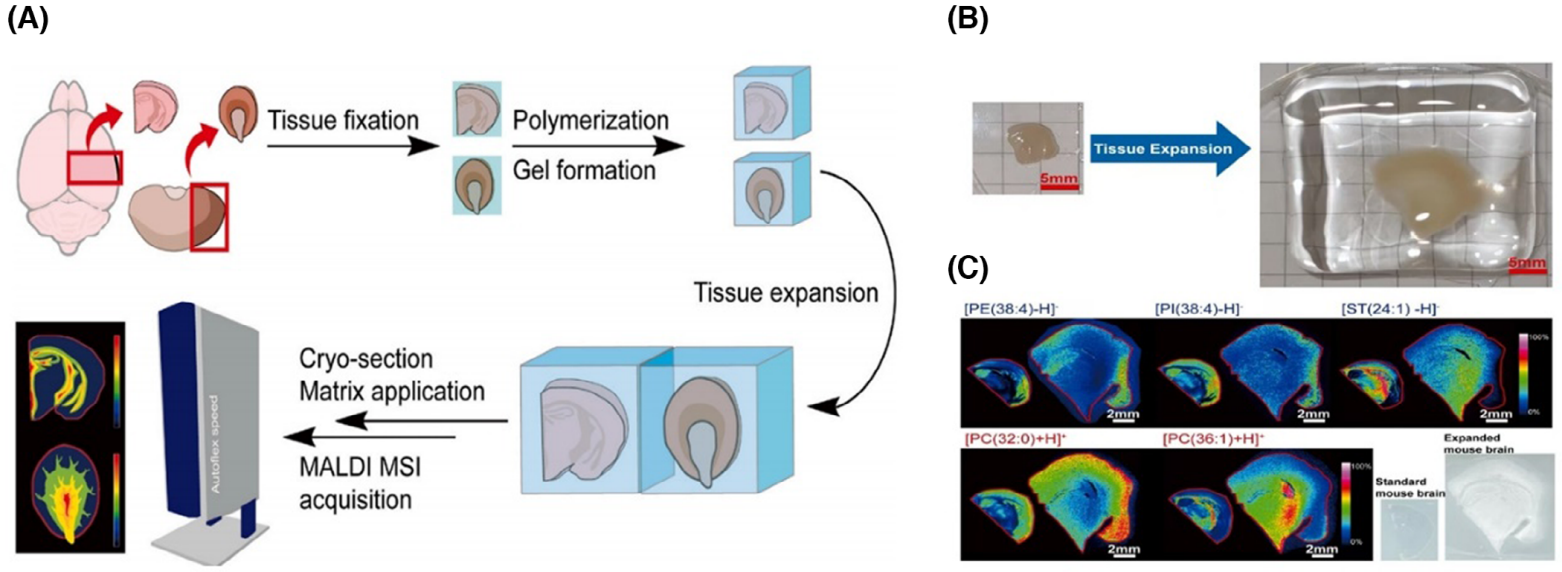
MSI expansion experiment process and data collection.^62^ (A) Tissue expansion workflow for MALDI MSI. Samples were fixed, then polymerised in a cubic acrylamide gel and imaged by MALDI. (B) Expanded mouse brain tissue. (C) Comparison of MALDI MSI of mouse brain tissue before and after expansion.

At present, some studies have further improved MALDI, Chen et al.^62^ successfully combined amplification microscopy with MSI and designed a MALDI‐MSI compatible tissue amplification protocol, which can help to further improve the spatial resolution of MSI (Figure 3).

MALDI‐MSI exhibits excellent resolution and throughput, with a maximum resolution of 1–2 µm^63^ and can even reach the level of analysing individual cells. But MALDI‐MSI cannot provide immediate or in vivo metabolic information, and the matrix‐coated samples are not suitable for secondary analysis. In spite of some limitations, MALDI‐MSI is a technology with broad application prospects in the field of tissue metabolomics and spatial metabolomics.

#### 3.3.3 Desorption electrospray ionisation mass spectrometry imaging

DESI represents the inaugural instance of a spray‐based surface analysis method, which has since become a prevalent ambient ionisation technique. It guides the charged droplets and solvent ions from the electrospray to the surface of the sample to be analysed without requiring a matrix for the experiment (Figure 2F).^64^ The DESI method can be completed under atmospheric pressure without the limitation of vacuum conditions, using a pneumatic electric spray is used to directly act on the tissue surface of interest. This method is particularly advantageous because it can directly analyse samples without needing a harsh environment and has simple sample preparation. Additionally, it permits soft ionisation of biomolecules, including lipids and proteins. DESI utilises the same spray solution to obtain MS data in both positive and negative ion modes, which enhances efficiency and minimises tissue loss to the sample, but it needs to be properly optimised according to the analysis objectives and sample characteristics.^53^ Another advantage of DESI is that it allows in vivo sampling on the surface of living tissues. Although SIMS and MALDI provide histochemical images with high sensitivity, specificity and spatial resolution, they are invasive techniques and are not suitable for in vivo testing in a clinical setting.^65^ DESI has developed systems suitable for high‐throughput data analysis that improve on the imaging‐based approach by addressing issues such as lost sample processing time, reducing redundant information after single‐sample scanning, saving analysis time and accounting for the effect of isochronous progressive grid motion that characterises tissue imaging experiments.^66^

Spatial metabolomics with DESI‐MSI has been further enhanced by AFAI‐MSI technology. The system provides improved detection sensitivity, while expanding sample space and operational flexibility, making it suitable not only for single tissue samples but also for remote ion acquisition and imaging analysis of large volume samples.^67^ The total advantages of DESI are reflected in the simplicity of experimental operation. However, there remains a significant challenge in optimising DESI to increase sensitivity and ensure that the final image is not distorted and has a high spatial resolution.^68^

## 4 SPATIAL OMICS IN DISEASE RESEARCH

### 4.1 Advances in ST in current atherosclerosis‐related studies

ST is applicable to three biological problems: ST approaches can clarify the cell type make‐up of tissues, cellular interactions are relevant and they can be an aid in the elucidation of molecular interactions between tissue components.^69^ The scRNA‐seq has been increasingly adopted in the field of transcriptomics research since it development. It allows for the analysis of the whole transcriptome at a single‐cell resolution, enabling in‐depth characterisation of cellular heterogeneity in tissues. However, the inability of scRNA‐seq to be performed at in situ sites results in a deficiency in information about the interactions and tissues of the studied objects in the tissue field of view. While ST can provide quantitative gene expression data and micro‐angled visualisation of mRNA distributions within tissue sections, and enable bioinformatic analyses, which are of great value in research and diagnostics.^70^

Presently, ST serves as an advanced tool in the study of atherosclerosis. The spatial localisation of different cell types within atherosclerotic plaques can be investigated using ST, including the location of smooth muscle cells (SMCs), macrophages and endothelial cells, as well as their functional state. Kawai et al.^71^ analysed 3842 cells from two eroded plaques for gene expression and ACTA2 transcript expression. ST of gene expression was analysed in clonal and non‐clonal regions using BaseScope technology to investigate transcriptomic differences in targeted regions of the SMCs in plaque erosion region. The results showed that ACTA2 had high expression in clonal regions, while CD74, SERF‐2, CTSB and HLA‐DPA1 had low expression.^71^ It is noteworthy that both clonal and nonclonal cellular plaques were present in unstable plaques. However, polyclonal cells primarily constituted the intimal plaque lesions, with monoclonal cells accounting for only a minority. The presence of both clonal and nonclonal SMC regions in atherosclerotic plaques suggests that SMCs proliferate in a heterogeneous manner. This finding further validates the hypothesis that SMCs populate plaques in a manner distinct from that of so‐called atherosclerotic stem cells.^72^

Another recent study, Sun et al.^73^ applied Visium ST to detect gene expression and pathway‐specific spatial locations in human plaques. They validated pathways previously associated with the proximal rupture‐prone region and located mRNA levels of MMP‐9, IGKC and PLN within a single plaque tissue section.^73^ This new technology shows potential in the field of atherosclerosis. In addition, plaque rupture is an infrequent occurrence in the marginal region of the stenotic centre. Instead, rupture sites exhibit distinctive gene expression patterns that are most pertinent to atherosclerosis‐related illnesses. This enhances our comprehension of the molecular factors associated with plaque rupture and localises the expression of specific genes.

Furthermore, Ravindran et al.^74^ validated the TRAP‐Seq findings through histological and ST analyses of human carotid artery and mouse tissue sections. They observed that Itih4 was specifically expressed in SMCs within atherosclerotic lesions. Interestingly, they found that tih4 is also associated with coronary artery disease by targeting it for genome‐wide association studies, co‐localisation of splicing quantitative trait loci (QTL) and protein QTL signals.^74^

ST is a highly effective method for capturing all polyadenylated transcripts, thereby facilitating the visualisation of the expression of numerous genes of interest in raw tissue sections, which will greatly assist us in obtaining deep information about tissues. Moreover, it enhance our ability to gain deeper insights into biological processes.

### 4.2 Advances in spatial proteomics in current atherosclerosis‐related studies

Spatial proteomics is the study of protein subcellular distribution and abundance.^75^ The organism tightly regulates the subcellular localisation of proteins, which is intimately linked to their function. Therefore, understanding how proteins localise and distribute at the subcellular level is vital to a comprehensive of cellular biology. Traditional proteomics primarily focuses on protein expression level and function, but this approach loses spatial distribution and cell type information during tissue homogenisation. As a result, MS signals only provide the average level of protein expression in tissue or cell lysates. The field of spatial proteomics aims to link spatial and cell type information with proteomic data to gain insights into the spatial microenvironment of tissues and discover more precise biomarkers and novel functional mechanisms.

Spatial proteomics has yet to be explored in the field of atherosclerosis. However, we were able to identify a number of proteins from traditional proteomics studies that are relevant to understanding disease. These components could serve as targets for future spatial proteomics studies. Tan et al.^76^ were the first to establish the connection between serum MMP‐9 levels and the stability of carotid plaques, identifying MCP‐1 and MMP‐9 as key mediators of AS. A comprehensive understanding of these proteins may facilitate the development of novel intervention strategies to mitigate the formation and rupture of unstable atherosclerotic lesions.

Bhosale et al.^77^ conducted an evaluation of serum protein biomarkers associated with the early stages of carotid atherosclerotic plaque formation in humans. Their cohort measurements demonstrated that distinct proteomic profiles were evident in subjects exhibiting indications of plaque development.^77^ Among these profiles, FBLN1C was identified as being associated with the plaque extracellular matrix. These findings suggest potential targets for further studies of protein interactions. Nevertheless, additional studies about FBLN1C are currently lacking.

Baragetti et al.^78^ extended the findings of previous serum protein studies, including polymeric immunoglobulin receptor, chemokine ligand 18, carbonic anhydrase 1, Fc gamma receptor IIa and reduced matrix metallopeptidase 10, gastrotropin, interleukin 7 receptor, were identified as possible markers of subclinical carotid atherosclerosis development. The study focuses on proteins and peptides that are strongly associated with the disease, such as C‐type natriuretic peptide,^79^ latent transforming growth factor β‐binding protein 1,^80^ bilirubin,^81^ myeloperoxidase^82^ and related proteins involved in functions and pathways related to iron death and lipid metabolism.^83^ Spatial proteomics can be used to further elucidate the role of these substances in the disease mechanism. Future exploration in this area is promising.

**FIGURE 4:**
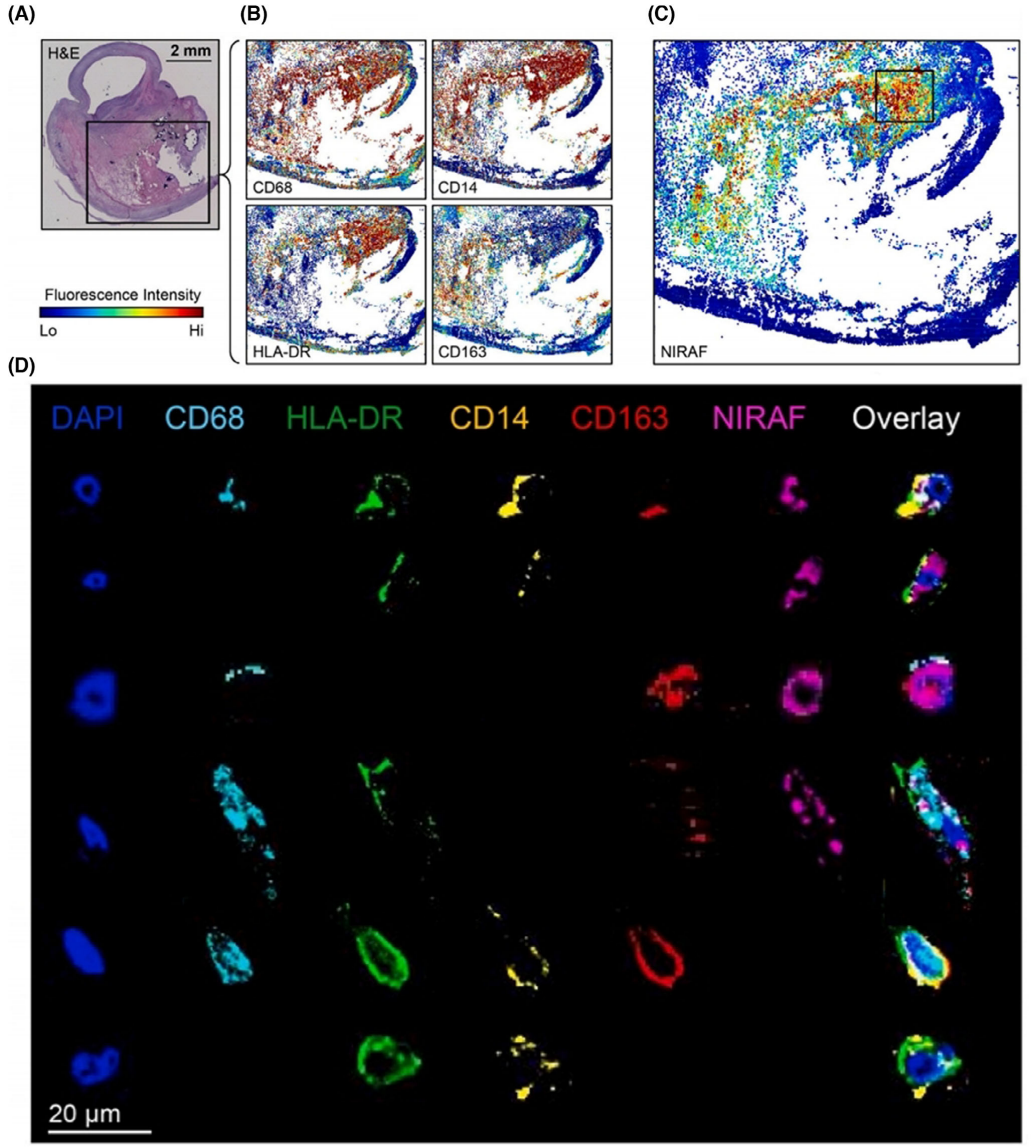
Spatial proteomics analysis of isolated single cells with CODEX.^84^ (A) H&E cross‐section of plaques. (B) CD68, CD14, HLA‐DR and CD163 localisation on CODEX in same section. (C) NIRAF signal intensity on the same tissue section in CODEX images. (D) Representative fluorescent samples of manually segmented individual cells from CODEX.

The preliminary work on related spatial omics is now being conducted by researchers. Schneider et al.^84^ combined ST and spatial proteomics with CODEX imaging to explore the potential of NIRAPA‐ultrasound imaging in conjunction with IHC for the detection of vulnerable carotid plaques, reveal that spatial correlation of CD74, HLA‐DR, CD14 and CD163 markers with disease (Figure 4). The specific therapeutic strategy required is contingent upon the characteristics of the plaque in question, with a range of options available, including intensive pharmacological treatment and surgical intervention. It is therefore imperative that such biomarkers are identified, as this will facilitate the development of more precise medicine and personalised disease assessment and treatment, based on spatial omics findings.

### 4.3 Advances in spatial metabolomics in current atherosclerosis‐related studies

The continuous development and improvement of MSI has greatly aided carotid atherosclerosis research. MSI provides spatial information on samples of increased biocomplexity with its high sensitivity, high throughput, high specificity, high reproducibility and wide dynamic range, which is the most common technique for obtaining spatially dimensional data.^85^ Some studies have been carried out to explore atherosclerotic plaques from various aspects of atherosclerotic plaques using the TOF‐SIMS,^55^, ^86^, ^87^ MALDI‐MSI^88^, ^89^, ^90^, ^91^, ^92^ and DESI‐MSI^65^, ^93^, ^94^ techniques to explore atherosclerotic plaques from various aspects. According to its characteristics, MSI has unique advantages in the detection of small molecules.^95^ Within biological tissues, spatial metabolomics offers a direct means of obtaining information on the structure, content and spatial distribution of a multitude of molecules.

The analysis through MSI is conducted in three principal dimensions: qualitative, quantitative and localisation. This breakthrough solves the issue of spatial information loss in traditional metabolomic research. For instance, Moerman, Astrid et al.^92^ used MALDI‐MSI studies to clarify that the concentrations of oxidised cholesteryl ester species and sphingomyelin (SM) were raised in necrotic intima areas, whereas diacylglycerol and triacylglycerol (TG) showed a spatial correlation with the zones that included the coagulation protein fibrin. Manicke et al.^65^ demonstrated that the lipid composition of plaques can vary between different specimens and also undergoes alteration during the progression of the disease. Li et al.^93^ collected carotid atherosclerosis specimens from 12 patients and utilised DESI‐MSI to study the lipid distribution in different regions of carotid atherosclerosis and at different pathological stages. SM, ceramide (Cer), phosphatidylcholine (PC) and LysoPC may be potential targets for novel lipid‐lowering drugs in addition to cholesterol ester and TG.^93^ Moreover, the distribution of lipids and their associated metabolic pathways varied significantly across different regions, indicating that distinct metabolic mechanisms in these areas of the carotid artery might play a key role in the development of atherosclerosis. This study has intrinsic value, but the sample size is relatively limited. Consequently, the sample should be augmented in subsequent research to enhance the credibility of the data. Slijkhuis et al.^94^ used DESI‐MSI to accurately match 330 features *m*/*z* demonstrated the efficacy of matching spatial lipids to histological regions of interest. This approach revealed a number of molecular species that exhibited co‐localisation with relevant disease processes in plaques. These included sphingolipid and Cer species specific for the calcification of plaques, as well as phospholipids and free fatty acids (FFA) for the inflammation of plaques, and TGs and phosphatidylinositols that were present within regions that had a high fibronectin content. In a recent observational study, Cao et al.^96^ applied MALDI‐MSI to compare the metabolic differences between early and late case stages of atherosclerotic mice and normal mice. The study showed that lysophosphatidylcholine (LPC) and lysophosphatidic acid (LPA) were significantly elevated in late‐stage plaques.^96^ Additionally, higher levels of both lipids were also observed in human carotid atherosclerosis samples for comparison. They point that the changes in phospholipid metabolism can lead to imbalances in energy metabolism and directly contribute to atherogenesis.

Furthermore, Greco et al.^58^ performed a multimodal data analysis comparing lipid profiles of specific histopathologic regions within plaques. The results showed that symptomatic plaques’ macrophage‐rich regions were characterised by an enrichment of sphingolipids, as well as by the presence of cholesterol and cholesteryl ester‐rich endothelial SMCs. The ability of MALDI‐MSI to analyse specific regions of atherosclerotic lesions was revealed to be a promising technique for researching histologically heterogeneous atherosclerotic plaques.

## 5 LIMITATIONS AND CHALLENGES

**FIGURE 5:**
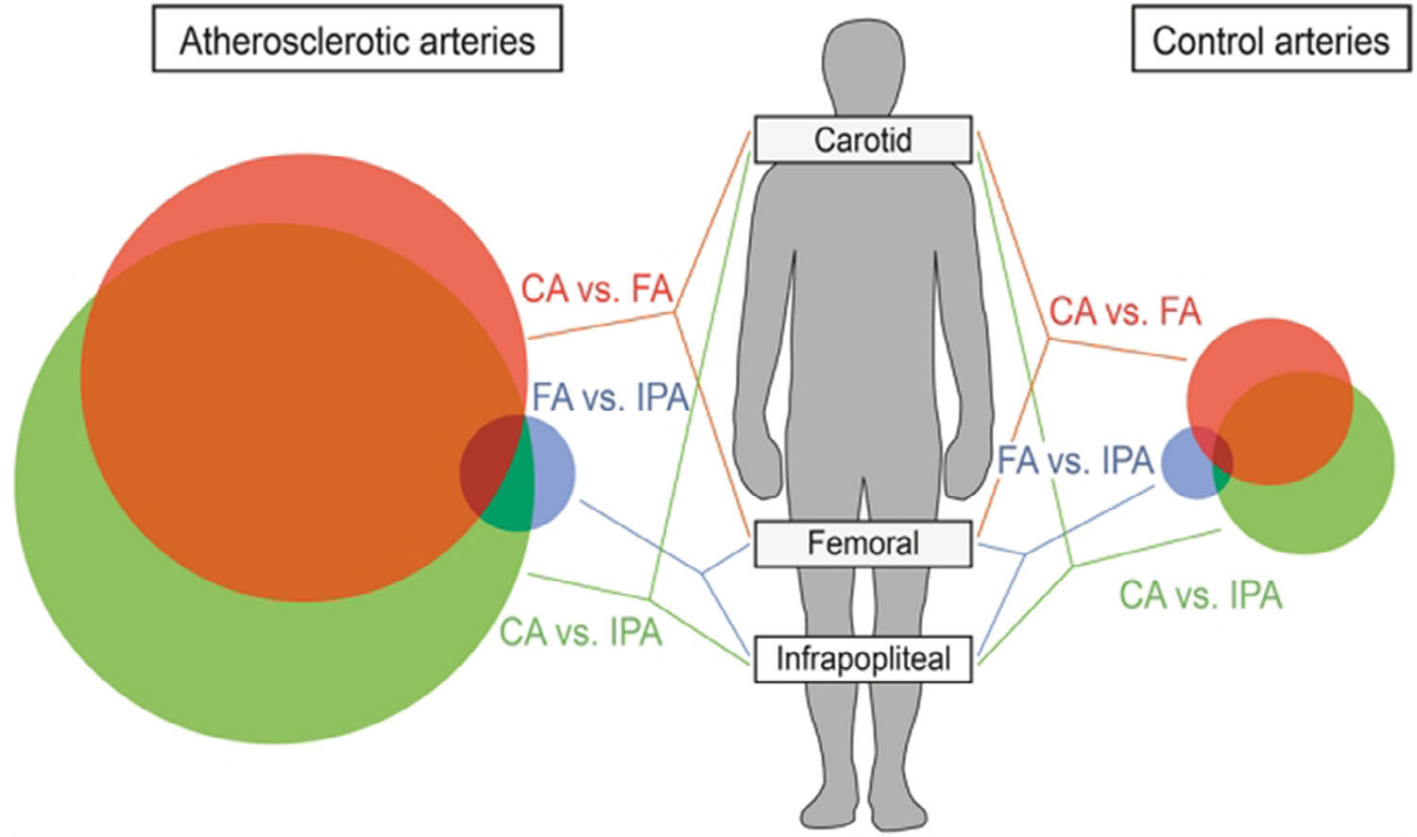
Venn diagram of the genes that are differentially expressed in three arterial regions associated with atherosclerotic and healthy arteries.^106^

The plaque samples obtained and utilised in the current research project were derived from carotid endarterectomy, which represents the primary source of data and information collection in the majority of contemporary studies. However, it should be noted that atherosclerotic disease does not only occur in the carotid arteries, but also in the coronary arteries, femoral arteries and other vessels. While AS is the underlying pathology, some researchers have observed that the progression of AS not only varies significantly between individuals but also shows specific heterogeneity in different arterial beds.^97^, ^98^, ^99^ Spatial omics studies are also being conducted in different vascular beds than the carotid arteries, and MSI have been extensively utilised (Table 3).^55^, ^86^, ^87^, ^88^, ^100^, ^101^, ^102^, ^103^, ^104^, ^105^ Furthermore, there is even a difference in the characterisation of the arterial beds at the genetic level (Figure 5).^106^, ^107^ For instance, Sukhanov et al.^108^ used ST to identify global transcriptomic alterations in advanced plaque. This approach was used to gain insights into the mechanistic consequences of IGF‐1. ST analysis revealed that IGF‐1 inhibited gene expression of FOS/FOSB factors, as well as MMP9 and CXCL14 in plaque macrophages, these molecules may be involved in the IGF‐1 effect on atherosclerosis. Interestingly, the use of ST showed that IGF‐1 induced major significant changes in the plaque transcriptome, and the authors found that IGF‐1 was able to counteract atherosclerosis to promote plaque stabilisation and reduce endothelial cells damage in experiments in the FH pig model. We believe that expansion of data collection and control studies to multiple vascular beds promises in‐depth evaluation of specific mechanisms.

**TABLE 3:** Application of MSI in spatial omics studies of other vascular beds.

Technology	Sample	Arterial bed	Years	References
MALDI‐MSI	Human/Mouse	Femoral artery/aortic roots	2011	^88^
TOF‐SIMS	Mouse	Aortic sinuses	2013	^55^
TOF‐SIMS	Human	Coronary artery	2015	^86^
MALDI‐MSI	Human/Rabbit	Aorta/ascending aorta	2015	^100^
MALDI‐MSI	Mouse	Aortic roots	2020	^101^
MALDI‐MSI	Rabbit	Thoracic aorta	2020	^102^
TOF‐SIMS	Human	Coronary artery	2021	^87^
MALDI‐MSI	Human/mouse	Lower extremity artery/aorta or carotid artery	2022	^103^
MALDI‐MSI	Human	Coronary artery, carotid sinuses	2023	^104^
MALDI‐MSI	Swine	Coronary artery	2024	^105^

At present, data pertaining to spatial omics are predominantly derived from slices. However, the use of these slices in imaging experiments may result in tissue defects, thereby affecting subsequent processing and the accuracy of imaging comparisons. As spatial technology advances, the challenge remains to develop methods for acquiring both histology and histology information from the same tissue section simultaneously, as well as to enhance the scalability of spatial analysis techniques.

## 6 CONCLUSIONS AND FUTURE DIRECTIONS

To date, spatial omics is a novel concept, and its development has deepened the understanding of atherosclerotic plaques, particularly in exploring potential therapeutic targets, identifying disease pathways and facilitating clinical risk assessment. This progress provides a theoretical basis and is now a crucial tool for basic research in this field. As mentioned earlier, spatial omics can be represented by a variety of experimental assays, opening new avenues for biomarker discovery and providing more scientific guidance for hypotheses in mechanistic studies. At present, original research is beginning to revisit the molecular distribution in atherosclerotic plaques using the concept of spatial omics. However, there is a lack of more fundamental research using spatial omics on the deeper mechanisms associated with carotid atherosclerosis causing stroke and how to comprehensively assess molecular distribution. Although there is a growing body of work applying spatial genomics concepts to spatial molecular studies of disease, there is no detailed summary of the existing work.

The advancement of sophisticated MSI techniques and spatial omics technologies, including spatial analysis of chromatin accessibility, promises a significant step forward in the construction of a spatial atlas of human atherosclerosis. The inability to confidently annotate peaks associated with studied metabolites can hinder our ability to identify key metabolites in plaques. Therefore, it is important to develop technological innovations that improve the currently available high‐confidence peak annotation platforms for future studies, as well as refine the different spatial profiles and comprehensive annotation.^104^

Spatial multi‐omics has become a promising approach to comprehensively analyse the spatial distribution of molecules in tissues, capable of representing the visual structure of tissues in multiple data modalities such as transcriptome, epigenome, proteome and metabolome by jointly analysing them simultaneously or even in the same tissue section and displaying localisation information by constructing a 3D map of molecules can be a valuable method to advance research. However, spatial multi‐omics also requires more detailed experimental design, and improvement in sample preparation protocol is essential for the successful execution of experiments as well as for boosting the reliability of the data obtained. Particularly in the context of tissue samples, which must adhere to established protocols for tissue sampling, fixation, freezing and sectioning to enhance experimental efficiency. Experimental results from spatial multi‐omics studies can generate large datasets that require significant data processing. Implementing high‐throughput screening analyses can enhance the efficiency of storing and managing the data. No matter how it is undeniable that the expected future advances in spatial omics include improvements in throughput, cost reduction and increases in sensitivity and specificity, as well as the integration of more modes of analysis into a single assay,^109^, ^110^, ^111^ we believe that the development of MSI will be an important supporting technology for future spatial omics research. Considering the preciousness of human tissue samples, it becomes crucial to maximise the information obtained from a single analysis, so in the future, we should consider joint multimodal analyses, which will facilitate a large amount of information obtained from experimental data. It is worth noting that although a large number of new research strategies have been reported, they have their own advantages and are not universally applicable. The selection of an appropriate research method is contingent upon the nature of the substances under investigation, which may include factors such as molecular size, molecular species and molecular ionisation properties. Prior to embarking on experimentation, it is essential to consider the most suitable research method for the given substances.

Current research indicates that the fusion of spatial omics has greatly facilitated the study of atherosclerosis. This technique allows for the spatial visualisation of plaques and revisiting potential targets and mechanisms of action of the disease. This direction can aid in the early diagnosis and prognosis of atherosclerosis, as well as identifying potential biomarkers for a better understanding of its pathophysiology. We believe that, with the aid of emerging spatial omics, we will be able to gain a deeper understanding of atherosclerosis in the future.

## AUTHOR CONTRIBUTIONS

**Jiaxin Wan**: Writing—original draft, investigation, visualisation, editing and proofreading. **Zhi Sun**: Writing—review and editing, conceptualisation, supervision. **Xueqiong Feng, Mateus T. N. Macho, Peipei Zhou, Zhouyang Jiao, Hui Cao Chuang Zhang, Rijin Lin, Xiaowen Zhang**, **Mengyan Fan, Nan Zhang**, **Jiamei Zhang, Huixiang Liu**: Writing—review and editing. **Jing Li**: Writing—review and editing, project administration, funding acquisition, supervision, conceptualisation. **Sheng Guan**: Writing—review and editing, funding acquisition, supervision.
